# Translational Inhibition of α-Neurexin 2

**DOI:** 10.1038/s41598-020-60289-8

**Published:** 2020-02-25

**Authors:** Xiaoting Ding, Shasha Meng, Jiahong Zhou, Juan Yang, Hongmei Li, Weihui Zhou

**Affiliations:** 1 Ministry of Education Key Laboratory of Child Development and Disorders; Chongqing Key Laboratory of Translational Medical Research in Cognitive Development and Learning and Memory Disorders, National Clinical Research Center for Child Health and Disorders, China International Science and Technology Cooperation base of Child development and Critical Disorders, Children’s Hospital of Chongqing Medical University, 136 ZhongshanEr Lu, Yuzhong District Chongqing, 400014 China; 2 Dermatology Department of Children’s Hospital of Chongqing Medical University, 136 ZhongshanEr Lu, Yuzhong District Chongqing, 400014 China

**Keywords:** Translation, Molecular neuroscience

## Abstract

Neurexins are extensively investigated presynaptic cell-adhesion molecules which play important roles in transmitting signals and processing information at synapses that connect neurons into a vast network of cellular communications. Synaptic transmission of information is a fast and dynamic process which relies on rapid and tight regulation of synaptic protein expression. However, the mechanism underlying those regulation is still not fully understood. Therefore, we explore how the expression of *NRXN2α*, one of encoding genes for neurexins, is regulated at the translational level. *NRXN2α* transcript has a long and conserved 5′-untranslated region (5′UTR) suggestive of the rapid regulation of protein expression at the translational level. We first demonstrate that the 5′UTR has negative effects on the expression of the *NRXN2α* and find a critical subregion responsible for the major inhibitory function. Then we identify a particular secondary structure of G-quadruplex in the 5′UTR. Moreover, we find that the synergistic roles of G-quadruplex and upstream AUGs are responsible for most of NRXN2α-5′UTR inhibitory effects. In conclusion, we uncovered 5′ UTR of neurexin2 potentially inhibits neurexin2 translation by multiple mechanisms. In addition, this study underscores the importance of direct protein quantitation in experiments rather than using mRNA as an indirect estimate of protein expression.

## Introduction

Synapses are connective structures where billions of neurons communicate with each other and transfer information from a presynaptic neuron to a postsynaptic cell. The high specificity of neurons and complex synaptic connections are the basis of brain function. Synaptic transmission of information is fast, efficient, dynamic and tightly controlled^1,2^. A synapse is made up of a presynaptic and postsynaptic terminal. At the synapse, the presynaptic terminal secretes neurotransmitters to the postsynaptic terminal for the transmission of information between the presynaptic neuron and the postsynaptic neuron. There are many distinct cell adhesion molecules located in presynaptic terminal membrane and postsynaptic terminal membrane respectively. Synaptic junctions are thought to be organized by trans-synaptic cell adhesion molecules, which coordinate synapse formation, restructuring, and elimination in both directions^2,3^.

Neurexins, a group of presynaptic proteins encoded by *NRXN* genes, are crucial synaptic cell adhesion molecules which mediate synaptic plasticity and maturation of chemical synapses^1,4^. The mammalian genome contains three *NRXN* genes (*NRXN1, NRXN2* and *NRXN3*). Each encodes an α-protein and a β-protein from two independent promoters^5^. Previous studies reported that α-neurexin can bind to CASK, Munc18, syntenin and synaptotagmin, presenting a crucial role in vesicular release^6,7^. By postsynaptic binding with leucine-rich repeat transmembrane proteins (LRRTMs) or dystroglycan, α-neurexin can directly influence NMDA, AMPA or GABAergic receptors at the synapse, thereby altering a cell’s excitatory or inhibitory ability^8–11^. Although the function of neurexins are important for the formation and transmission of synapses, previous studies on the mechanisms of its expression regulation mainly focused on post-transcriptional splicing regulation^12,13^, and little is known about other types of expression regulation. Recent studies have shown that the pattern in mRNA expression of *NRXNs* is significantly different from that of proteins. For example, the qPCR detection of *NRXNs* mRNA in different brain regions of mice after 30 days of birth shows that the levels of α-transcripts are 10–100 times higher than that of β-transcripts in general. Among these mice the mRNA level of *NRXN2*α in cerebellum is 100 times higher than that of *NRXN2β*^14^. However in terms of protein levels, the α-proteins are only 2–4 times higher than β-proteins in general, and in the cerebellum, there is even more expression of β-neurexin2 than that of α-neurexin2^15^. The mechanism and significance of this phenomenon are unclear.

In eukaryotes, regulation of protein expression is controlled at multiple levels, such as transcription, posttranscriptional processing, translation, posttranslational modification, and protein clearance. Among them, translational regulation is a major determinant of protein production rates. The untranslated region of mRNA, especially the 5′UTR, is the key mediator for protein translational regulation and contains many cis-acting control elements^16^. These control elements in 5′UTR include the m7G cap, upstream AUGs (uAUGs), upstream open reading frames (uORFs), internal ribosome entry sites (IRES), secondary structure, length, nucleotide sequence context around the AUG start codon and some specific sequences as binding sites for regulatory proteins^17–19^.

In the present report, we have investigated the effects of NRXN2α-5′UTR on its protein translation and the mechanisms controlling *NRXN2α* translation. We first demonstrate that the NRXN2α-5′UTR has negative effects on the expression of the downstream gene and identified a particular G-quadruplex in the 5′UTR of *NRXN2α* mRNA. Moreover, we find that the synergistic roles of G-quadruplex and uAUGs are responsible for most of 5′UTR inhibitory effects. Our results explain the possible mechanisms by which mRNA expression of *NRXN2α* is much higher than that of *NRXN2β* in different brain regions, but protein expression is not.

## Results

### Sequence analysis of human *NRXN2α* mRNA 5′UTR

**Figure 1:**
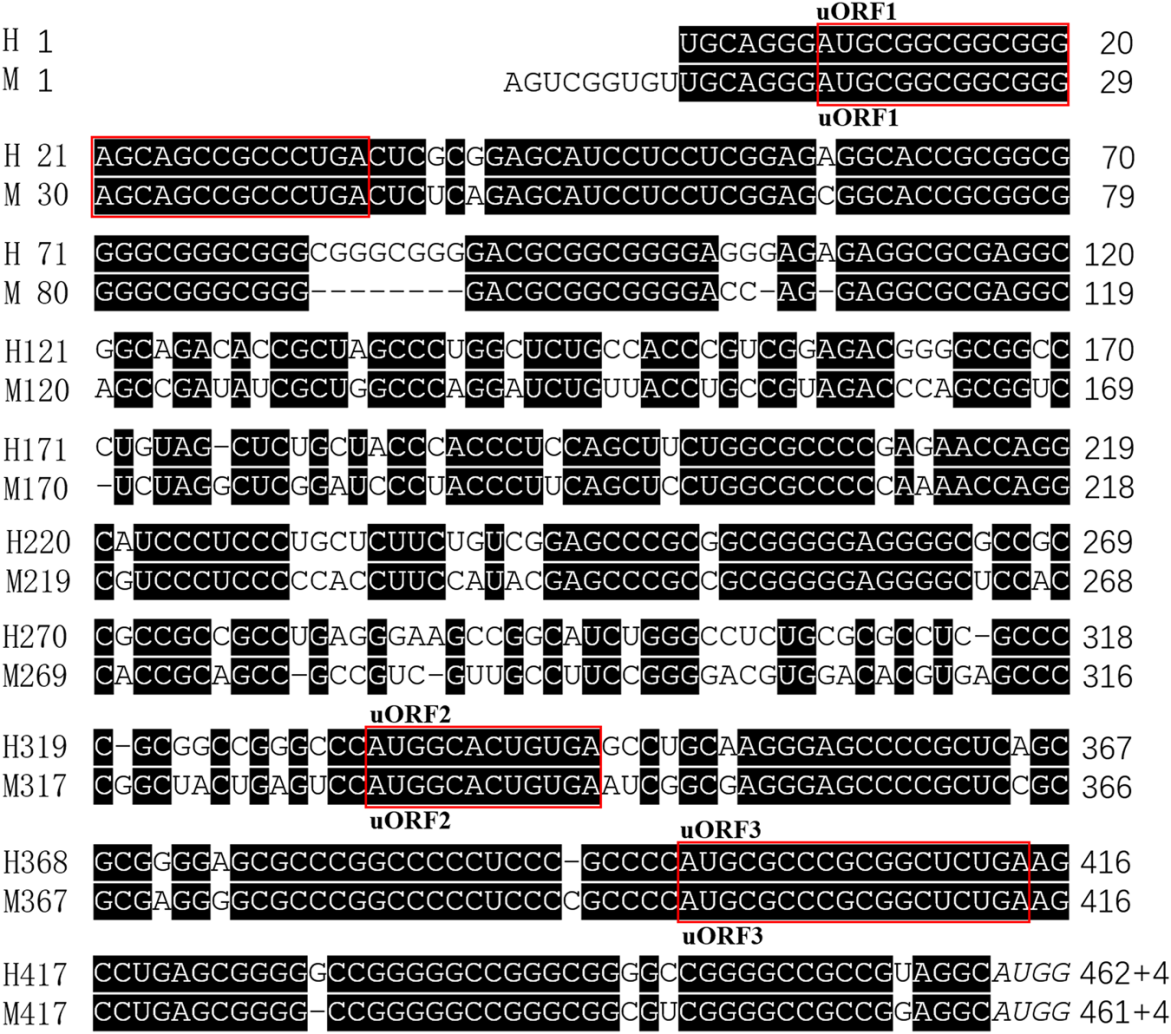
The 5′UTR of *NRXN2α* is conserved between human and mouse and has multiple potential elements for regulations. The nucleotides shown on the black background are consistent between the human and the mouse and the sequence identity between them is 76%. The uORFs are boxed by the red rectangles. The last AUG is the translational starting site of main ORF of *NRXN2α* mRNA. H and M represents human and mouse, respectively.

The reference sequences of human *NRXN2α* and mouse *Nrxn2α* gene mRNA were extracted from NCBI. There are two reference sequences for human *NRXN2α* gene mRNA, namely NM_015080.3 and NM_138732.2, which have completely consistent 5′UTR sequences. There are four reference sequences for moue *Nrxn2α* gene mRNA, namely NM_001205234.2, NM_020253.4, NM_001205235.2 and NM_001369363.1.All of them have consistent 5′UTR sequences. Sequence analysis of human *NRXN2α* gene mRNA revealed that it contains a long 5′UTR consisting of 462 nt that has a high GC content (79%), three uAUGs and three uORFs (Fig. 1). The three uORFs are exactly consistent between human and mouse. Furthermore, the sequences of 5′UTR is highly conserved between the human and the mouse with the sequence identity of 76% between two species. These characteristics indicate that it may play a role in regulating protein translation.

### 5′UTR of NRXN2α mRNA represses protein expression

**Figure 2:**
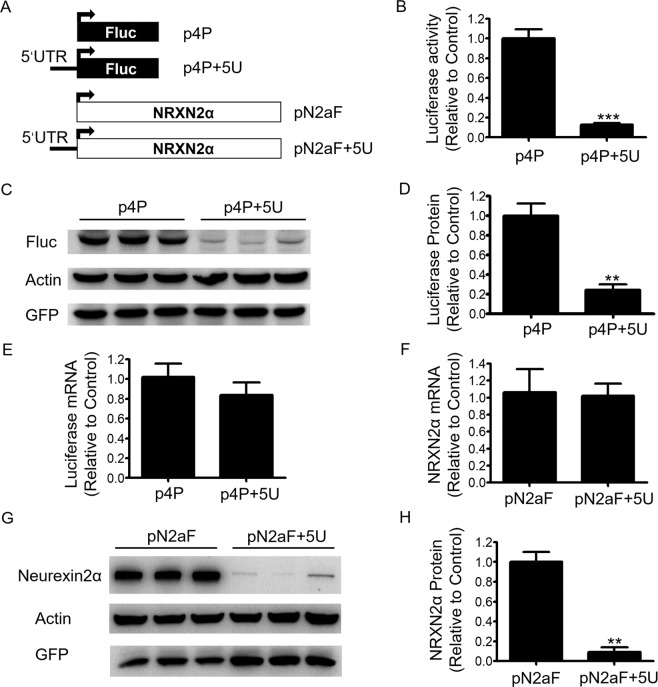
The 5′UTR of *NRXN2α* represses protein expression of downstream cistron. (**A**) Schematic diagram of plasmid constructs. The horizontal line represents the 5′ UTR of *NRXN2α*; the black rectangles indicate the open reading frame of firefly luciferase (Fluc) and the open rectangles indicate the open reading frame of human *NRXN2α* gene. (**B**) SH-SY5Y cells were co-transfected with luciferase reporters and p3PRluc. Firefly luciferase activity was measured 24 h after transfection, and Renilla luciferase activity was used to normalize for transfection efficiency. Data are shown as relative values over the samples transfected with empty control p4P plasmid. (**C**) Western Blot of luciferase protein. HEK293 cells were transiently co-transfected with luciferase reporters and EGFP expression plasmid. (**D**) Density analysis of the luciferase protein. The density was normalized to that of β-actin. (**E**) Quantification of the firefly luciferase mRNAs in SH-SY5Y cells transfected with reporter plasmids. The luciferase mRNA levels were normalized to β-actin mRNA levels. (**F**) Quantification of the *NRXN2α* mRNAs in HEK293 cells transfected with *NRXN2α* expression plasmids. The *NRXN2α* mRNA levels were normalized to β-actin mRNA levels. (**G**) Western Blot of neurexin2α protein expression. HEK293 cells were transiently co-transfected with *NRXN2α* and EGFP expression plasmid. (**H**) Density analysis of the neurexin2α protein. The density was normalized to that of β-actin. All values were expressed as means ± S.D., N = 3. ***P < 0.001 and **P < 0.01 by the Student’s *t* test. The full-length blots for (**C**,**G**) are presented in Supplementary Fig. 1.

First, to determine whether the 5′UTR of *NRXN2α* transcript can affect the translation of downstream cistrons, we constructed a reporter plasmid, named as p4P + 5U, by inserting the NRXN2α-5′UTR between SV40 promoter and the coding sequence of firefly luciferase on a blank control reporter plasmid p4P (Fig. 2A). SH-SY5Y cells were transfected with p3PRluc and either p4P + 5U or blank plasmid p4P. Results of dual-luciferase assay showed that the luciferase activity dramatically decreased in the cells transfected with the construct containing 5′UTR (p4P + 5U) compared with the cells transfected with the blank plasmid p4P (Fig. 2B). Detection of the firefly luciferase in the cell lysate by immunoblotting also revealed that the presence of the 5′UTR strongly reduced protein levels (Fig. 2C,D). To determine whether the decrease in luciferase activity and protein expression caused by the 5′UTR is due to the changes of transcription or translation, qPCR was performed to measure the reporter′s mRNA levels. The results showed no difference in the level of luciferase mRNA between the cells of the two groups (Fig. 2E), indicating that the 5′UTR did not alter the process of transcription, but did alter the process of translation.

To further confirm the inhibitory effect of the *NRXN2α*-5′UTR on its own protein expression and if such inhibitory effect is cell-type specific, HEK293 cells were transfected by *NRXN2α* expression plasmids with or without the 5′UTR. The results from Western blot showed a significant decrease of NRXN2α protein level in the presence of the 5′UTR compared with the plasmid lacking 5′UTR (Fig. 2G,H), even though mRNA level didn’t change (Fig. 2F). The above results demonstrate that *NRXN2α*-5′UTR has inhibitory effects on protein expression of downstream cistron.

### The uAUGs of the NRXN2α-5′UTR are partially responsible for reduced expression of NRXN2α

**Figure 3:**
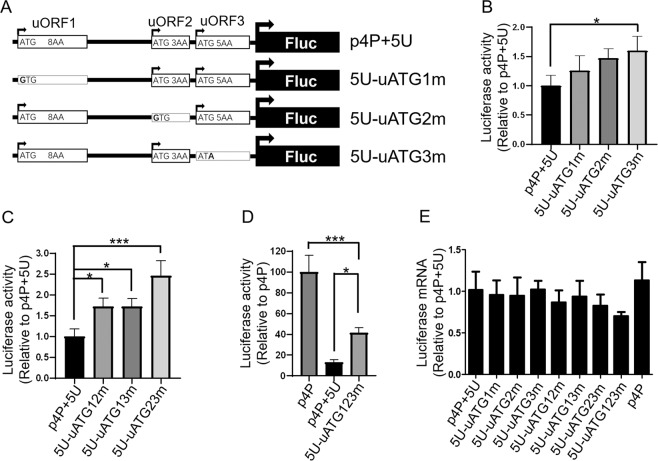
The uAUGs of the *NRXN2α*-5′UTR are partially responsible for reduced protein expression. (**A**) Schematic diagram of plasmid constructs. The black solid rectangle with the arrow to the right represents the luciferase open reading frame. The rectangular open box with the arrow to the right represents the uORFs and the number plus AA in the open box indicates the number of amino acids of uORFs. The rectangular open box without an arrow indicates that the ATG has been mutated to a corresponding non-ATG. (**B**) Comparison of luciferase activity of uATG single mutant in full-length 5′UTR in SH-SY5Y cells. (**C**) Comparison of luciferase activity of uATG double mutant in full-length 5′UTR in SH-SY5Y cells. (**D**) Comparison of luciferase activity of uATG triple mutant in full-length 5′UTR in SH-SY5Y cells. (**E**) Quantification of the luciferase mRNAs in SH-SY5Y cells that were transfected with corresponding plasmids. All values above were expressed as means ± S.D., N = 3. ***P < 0.001 and *P < 0.05 by One-Way ANOVA.

5′UTR of *NRXN2α* mRNA has three uORFs and three uAUGs (Fig. 1A). All three uORFs are highly conserved between the human and mouse, suggesting a biological function. Since both uAUG and uORFs in the 5′UTR are generally thought to inhibit the translation of the downstream main open reading frame^20–23^, we investigated the possible role of the three uAUGs or uORFs in the translational repression of downstream cistron by mutating those uAUGs and comparing various reporters’ activities using dual-luciferase report system. The mutation plasmids were constructed by mutating the start codon of the three uORFs from ATG to non-ATG (Fig. 3A) and then transfected into SH-SY5Y cells for 24 hours before measuring the luciferase activity. Mutation of uAUG3 increased luciferase activity of downstream reporter by 60%, whereas mutations of uAUG1 and uAUG2 showed a tendency to increase activity but were not statistically significant (Fig. 3B).

To explore the possibility of synergy between different uAUGs, the reporter plasmids with combined double mutations of the uATGs were generated. The results of luciferase assay show that each of the double mutation of uAUGs significantly increases luciferase activity compared with wild type. Furthermore, combined triple mutations at three uAUGs increased luciferase activity by about 320% and represented almost 42% of the total inhibitory capacity of full-length 5′UTR (Fig. 3D). Single and combined mutations of the uAUGs had no significant effect on the mRNA levels detected by qPCR (Fig. 3E).The above results show that uAUGs can inhibit the translation of downstream cistron, and the three uAUGs can work synergistically and are partially responsible for the inhibition of 5′UTR.

### The last third of the NRXN2α-5′UTR contains an inhibition element for translation

**Figure 4:**
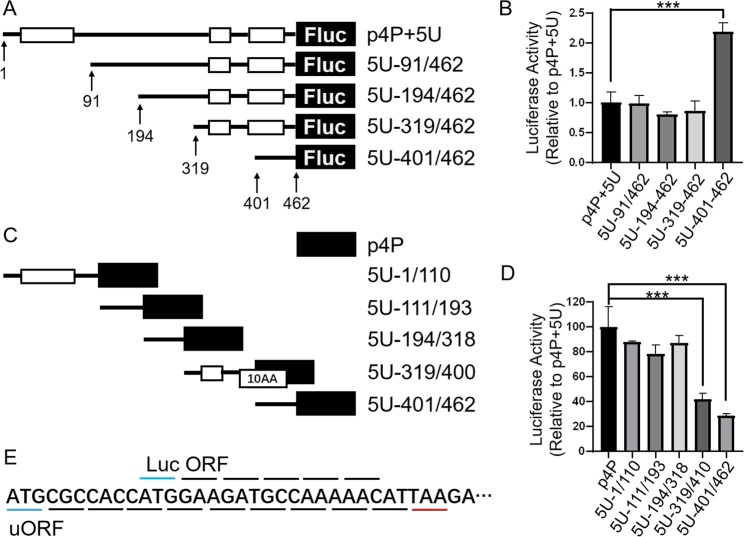
Effects of 5′UTR deletions on protein expression. (**A**,**C**) Schematic diagram of deletion plasmids. The open box with 10AA indicates an artificial uORF. (**B**,**D**) Comparison of luciferase activity of deletion mutant of 5′UTR in SH-SY5Y cells. (**E**) Sequence features of the artificial uORF and the part of luciferase ORF. The overlines correspond to the luciferase ORF, the underlines correspond to the uORF, the green lines indicate the start codons, and the red line indicates the stop codon of the artificial uORF. All values above were expressed as means ± S.D., N = 3. ***P < 0.001 by One-Way ANOVA.

Because uAUGs are only partly responsible for the inhibitory effects of full-length 5′UTR, in order to identify the other inhibitory factors, we truncated the 5′UTR to construct deletion plasmids for finding the functional regions inside 5′UTR. We first performed sequential deletion starting from 5′end of 5′UTR, transfected deletion plasmids into SH-SY5Y cells, and detected the luciferase activity. The results of luciferase assay show that sequentially deleting 91 nt, 102 nt and 124 nt from 5′end of 5′UTR 1–462 nt in plasmid p4P-5U (resulted in plasmids 5U-91/462, 5U-194/462, 5U-319/462, respectively) has no effects on luciferase activity (Fig. 4A,B).Further deletion of 81 nt (resulted in 5U-401/462) caused significant increase of luciferase activity compared to 5U-319/462 indicating that the 81 bp region between 319 and 400 have inhibitory function.

To further confirm the above results, the reporter plasmids containing dissected region of 5′UTR were constructed, and luciferase activity was detected. The results of luciferase assay show that dissected region of 1–110 nt, 111–193 nt and 194–318 nt have no inhibitory effects on luciferase activity (Fig. 4C,D) and the results are consistent with that of 5′end sequential deletion plasmids. Fragment of 319–462 nt maintained the full inhibitory capacity of the full-length 5′UTR and suggest that the right third of full-length 5′UTR is the main region of inhibition. When 319–462 nt is divided into two fragments of 319–400 nt and 401–462 nt, each fragment still maintained significant inhibitory effects even though each fragment no longer possessed the full inhibitory capacity of whole 5′UTR. Examination of this sequence show that uORF2 and uAUG3 are located in the region of 319–400 nt, and the stop codon of uORF3 is located in the region of 401–462 nt. The uAUG3 of 319–400 nt in plasmid 5U-319/400 could initiate an artificial uORF with only 10 amino acids, and the uORF was not in frame with the ORF of downstream luciferase (Fig. 4E). Taken together, the inhibitory effect of 5U-319/400 may come from the combined effect of uAUG2 and uAUG3. The region of 401–462 nt did not contain any uAUG or uORF. Although it was only 62 bp long, it still represents more than 50% of the inhibitory capacity of full-length 5′UTR.

### There is a G-quadruplex structure in the region of 401–462 nt in the NRXN2α-5′UTR

**Figure 5:**
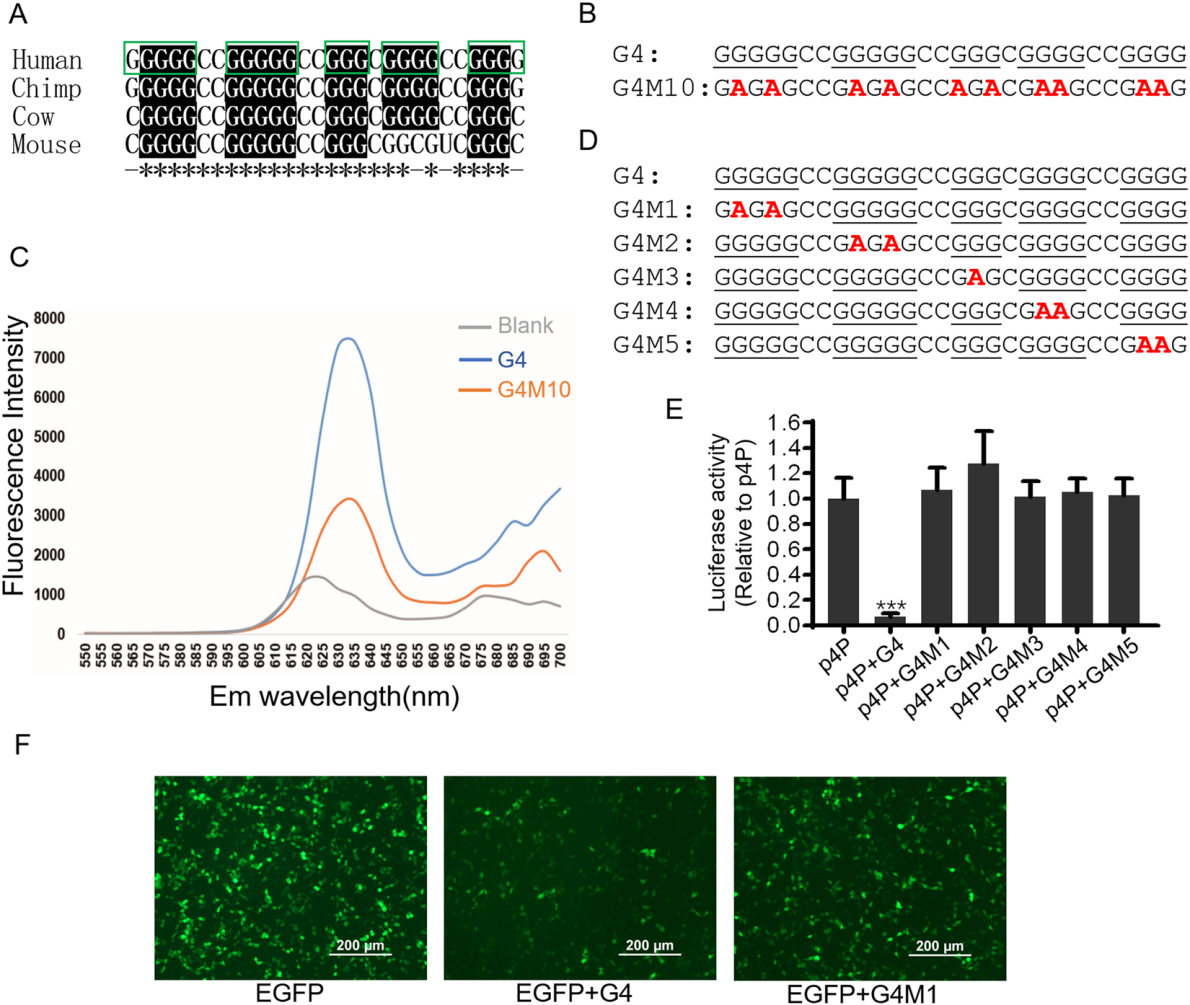
The identification of G-quadruplex and alignment of the G-quadruplex from four different species. (**A**) Alignment of the G4 sequences in *NRXN2α*-5′UTR from different species. The *indicates exactly the same sequences between different species; the nucleotides shown on the black background indicate conserved three G or four G repeats between species; the box with the green line represents the continuous G repeats in human. (**B**) The sequences of RNA oligos for PPIX binding assay. (**C**) Fluorescence emission spectra of PPIX binding assay. Blank represents the reaction of 1 μM PPIX dissolved in TE buffer (50 μMTris-HCl pH = 8.5) contained 100 mM KCl in the absence of RNA. (**D**) The sequences of G4 and corresponding mutants inserted into p4P. (**E**) Comparison of luciferase activity of G4 wild-type and mutant reporter plasmids in SH-SY5Y cells. Values were expressed as means ± S.D., N = 3. ***P < 0.001 compared to all other groups by One-Way ANOVA. (**F**) Effects of G4 wild type and mutant sequence on GFP expression.

Further analysis on the sequences of 401–462 region show that the GC content in this region was extremely high, up to 87.1%, and the content of G(33 nt) in this high GC region is much higher than that of C(21 nt) (Fig. 1). In addition, the ratio of GC content in the 424–451 region is 21G to 7C, and the distribution of G in this region presents multiple successive 3G or even 4G sequences. Moreover, the sequences between 424 and 451 nt were very conserved among species, suggesting biological functions (Fig. 5A).

DNA and RNA sequences with a minimum four “GG” repeats can potentially form stable G-quadruplex (G4) structure, a four-stranded conformation composed of stacked guanine tetrads (G-tetrads).The G-tetrads are formed when guanines are organized into planar quartets where each base is connected to two other bases by hydrogen bonds via the Hoogsteen faces of the guanine residues. Since highly structured G4 motif in 5′UTR has a high possibility to suppress the mRNA translation, in order to test whether this particular G4 structure can be formed *in vitro*, Protoporphyrin-IX (PPIX) binding assay was performed with synthesized RNA oligonucleotides (Fig. 5B) and PPIX. PPIX can specifically bind to the parallel G-quadruplex and present a significantly enhanced fluorescence signal. The results of PPIX binding assay show that the blank reaction without RNA have a low-fluorescence signal with emission peak at 624 nm, while the RNA oligos of 424–451 nt(referred to as G4 sequence) from 5′UTR of *NRXN2α* show significantly enhanced fluorescence signal, as well as a shift in emission peak from 624 nm to 635 nm.This indicates that the G4 sequence can form G-quadruplex *in vitro*. In contrast, the mutant(G4M10) of G4 sequence shows significantly reduced fluorescence signal (Fig. 5C).

To verify whether G-quadruplex can also be formed *in vivo*, we inserted the G4 sequence between the promoter and the luciferase ORF in plasmid p4P to construct the wild type G4 reporter vector(p4P-G4) and to test their effects on the expression of downstream cistron. The results of luciferase assay show that the inserted G4 sequence drastically reduces the luciferase activity (Fig. 5E). Since the formation of G-quadruplex usually requires only four continuous G repeats, there are five repeats consisted of continuous three Gs or four repeats consisted of continuous four Gs in the G4 sequence of *NRXN2α*-5′UTR. According to the prediction program of G-quadruplex(QGRS Mapper, http://bioinformatics.ramapo.edu/QGRS/index.php)^24,25^, there can be multiple combinations to form G-quadruplex. To determine which continuous Gs was involved in the formation of G4, we mutated each successive Gs on the basis of plasmid p4P-G4 (Fig. 5D). The results of luciferase assay show that the destruction of any continuous Gs leads to the complete loss of the inhibitory effect of the G4 sequence (Fig. 5E), suggesting that each successive Gs is critical for maintaining the inhibitory effect of the G4 sequence in the plasmid of p4P-G4. To further confirm the role of G4 sequence, we inserted the sequence of G4 or the mutant G4M1 between the promoter and the GFP ORF in vector pEGFPC2 to construct GFP expression plasmids. The results of cell transfection with these vectors show that G4 sequence can significantly reduce the expression of GFP protein, while the mutant G4M1 can reverse the inhibitory effect of G4 sequence (Fig. 5F).

### G-quadruplex and uAUGs play a synergistic role in the inhibitory effect of NRXN2α-5′UTR

**Figure 6:**
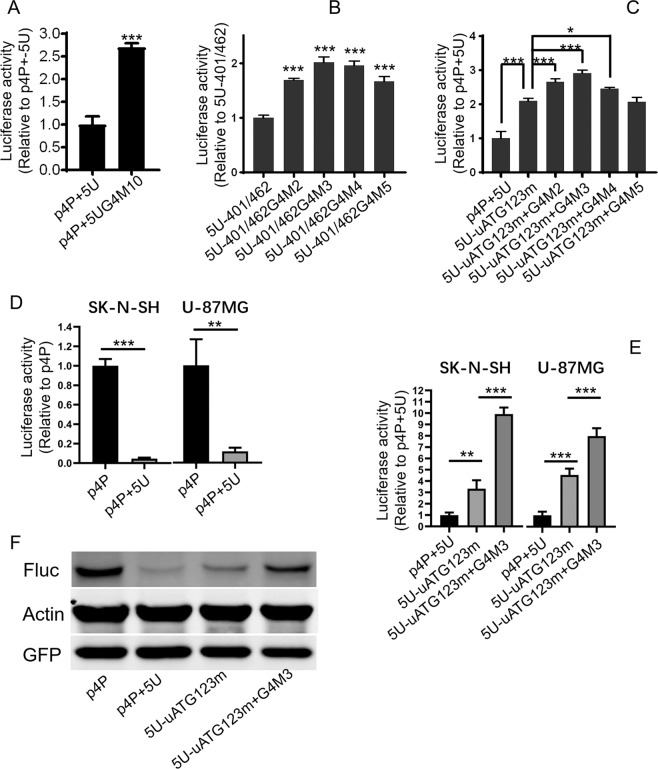
G-quadruplex and uAUGs played a synergistic role in the inhibitory effect of NRXN2α-5′UTR. SH-SY5Y cells (**A**–**C**), SK-N-SH and U-87MG cell (**D**,**E**) were co-transfected with various luciferase reporter plasmids and p3PRluc. Firefly luciferase activity was measured 24–48 h after transfection, and *Renilla* luciferase activity was used to normalize for transfection efficiency. Data are shown as relative values over the samples transfected with plasmids containing wild type sequences of *NRXN2α*-5′UTR or control plasmids. (**A**) Comparison of luciferase activity between reporter with wild type and G4M10 mutant of full-length 5′UTR. (**B**) Comparison of luciferase activity among reporter with wild type and corresponding mutants of 5′UTR-401/462 nt. (**C**) Comparison of luciferase activity among reporter with wild type, uAUG triple mutation only and combined mutations of uAUGs and G4 sequence in full-length 5′UTR. (**D**) Comparison of luciferase activity between reporter with wild type of full-length 5′UTR and control plasmids in SK-N-SH and U-87MG cell. (**E**) Comparison of luciferase activity among reporter with wild type, mutant with triple mutations of three uAUGs and mutant of both three uAUGs mutation and G-quadruplex mutation in full-length 5′UTR. (**F**) Western Blot of luciferase protein. U-87MG cells were transiently co-transfected with luciferase reporters and EGFP expression plasmid. All values above were expressed as means ± S.D., N = 3. ***P < 0.001, **P < 0.01 and *P < 0.05 by One-Way ANOVA (**B**,**C**,**E**) or *t*-test (**A**,**D**). The full-length blo*t*s for (**F**) are presented in Supplementary Fig. 2.

To further examine whether G-quadruplex in full length of 5′UTR still plays a role and whether G-quadruplex has a synergistic effect with uAUGs, the following experiments were conducted. The G4 sequence in the full length of *NRXN2α*-5′UTR in the plasmid p4P-5U was replaced by G4M10; the G4 sequence in the region 401–462 nt of *NRXN2α-*5′UTR in the plasmid 5U-401/462 was replaced by G4M2, G4M3, G4M4, G4M5,respectively; G4 sequence in the full length of *NRXN2α*-5′UTR with mutated three uAUGs in the plasmid 5U-uATG123m was replaced by G4M2, G4M3, G4M4, G4M5,respectively. Resulting plasmids and corresponding control were transfected into cells and luciferase activity was determined. The results of luciferase assay show that the mutation of G4 sequence in the full length of *NRXN2α*-5′UTR in the plasmid p4P-5UG4M10 can significantly reduce the inhibition of the whole length of 5′UTR (Fig. 6A). Consistent with the above results, mutations of G4M2, G4M3, G4M4, G4M5 in region 401–462 nt in the plasmids of 5U-401/462G4M2, 5U-401/462G4M3, 5U-401/462G4M4, and 5U-401/462G4M5 also significantly reversed the inhibitory effect of wild type sequences (Fig. 6B). Moreover, combined mutations of all three uAUGs and G4 sequence show a much weaker inhibitory effects compared with the mutation of only triple uAUGs (Fig. 6C). Taken together, the above results suggest that both uAUGs and G-quadruplex can exert inhibitory effects on downstream cistron respectively, and they can also exert stronger inhibitory effects synergistically.

To examine whether the 5′UTR in human *NRXN2α* gene mRNA can play a similar inhibitory role in cells from other human brain tissues, two cell lines, SK-N-SH and U-87MG, were transfected with reporter plasmids p4P and p4P-5U, respectively. The results of luciferase assay show that inserting 5′UTR of *NRXN2α* before the coding region significantly inhibits the expression of the downstream coding sequence in both cells (Fig. 6F). In order to verify whether uAUGs and G-quadruplex in the 5′UTR are responsible for its inhibition in these two cells, reporter plasmids p4P-5U, 5U-uATG123m and 5U-uATG123m + G4M3 were transfected into cells. The results of luciferase assay show that combined triple mutations at three uAUGs(5U-uATG123m) increases luciferase activity to about 3.3 folds in SK-N-SH and 4.6 folds in U-87MG, respectively (Fig. 6E). The mutations at three uAUGs plus G4M3 can further increase luciferase activity to 9.9 folds in SK-N-SH and 8.0 folds in U-87MG (Fig. 6E). To further validate these results, we detected the protein level of the firefly luciferase in the U-87MG cell transfected with reporter plasmids. The results from Western blot show a significant decrease of luciferase protein level in the presence of the 5′UTR(p4P-5U) compared with the plasmid lacking 5′UTR(p4P) (Fig. 6F). The reduced luciferase protein level in p4P-5U was partially reversed by plasmid with triple mutations of three uAUGs(5U-uATG123m), while the plasmid with triple mutations plus G4M3(5U-uATG123m + G4M3) almost completely reversed the luciferase protein level (Fig. 6F).This suggests that uAUGs and G-quadruplex are indeed responsible for the translation inhibition of 5′UTR in human *NRXN2α* mRNA and both elements can synergistically exert inhibitory effects on downstream cistron.

## Discussion

Phylogenetic Protein translation is a four-phase cyclical process consisting of initiation, elongation, termination and ribosome recycling^26^. Among them, the initiation is considered to be the most complex phase. There are two mechanisms to initiate the translation: canonical cap-dependent initiation and alternative cap-independent initiation^27^. Most eukaryotic mRNAs use cap-dependent scanning mechanisms, in which 43 s pre-initiation complex is recruited to the 5 prime end of the mRNA by eIF4 bound to the 5′-terminal m7G cap structure, scans in a 5′-to-3′ direction on the mRNA, and initiates translation at the first AUG^28^. The main difference between the cap-dependent and the cap-independent initiation is that the latter bypasses the requirement for cap structure and allows the 40 S ribosome to be directly recruited to the vicinity of the initiation codon by an IRES^27^. The regulation of protein translation occurs mainly at the initiation stage which is assumed to be the rate-limiting step of the translation. The 5′UTR, as the key mediator for initiation regulation, contains many unstructured and structured regulatory elements^25,29^. Generally, the average length of 5′UTR sequences roughly ranges from 100 and 200 nt, and the GC percentage is about 60%. However, the 5′UTR of *NRXN2α* is 462 nt long and the GC content is up to 79%. Moreover, the sequences of 5′UTR are highly conserved among different mammalian species and contain three uORFs. These features are strongly suggestive of a translational control of protein expression.

In this study, we first examined the effects of 5′UTR of *NRXN2α* on the main ORF of *NRXN2α* and the results show that the 5′UTR of *NRXN2α* dramatically inhibits the translation of *NRXN2α* (Fig. 2). Since uORF is generally considered to repress the translation of the downstream main ORF^30,31^, in order to clarify the mechanism of the inhibitory effect of *NRXN2α-*5′UTR, we analyzed and investigated the possible effects of the uAUGs in three uORFs on translation of downstream cistron. Although the fact that each single mutation at uAUGs mutated into non-AUG show a tendency to reduce 5′UTR’s inhibition, only uAUG3 mutation show statistical differences. Moreover, combined double or triple mutations of uAUGs show a markedly increase of luciferase activity compared with the wild type 5′UTR. Among them, the effect of double mutations at uAUG2 and uAUG3 was stronger than that of double mutations either at uAUG1 and uAUG2 or uAUG1 and uAUG3.Combined triple mutations at three uAUGs increased luciferase activity by 320% and represented almost 42% of the total inhibitory capacity of full-length 5′UTR. These results suggest that all uAUG are involved in inhibiting effect of the 5′UTR and uAUG1 seems to have the weakest contribution while uAUG3 seems to have the strongest contribution. The inhibition ability of uORF is influenced by many factors. Among them, whether uAUG can be recognized by ribosomes as a start codon is the basis of its inhibition, and whether it can be recognized as a start codon mainly depends on the nucleotide context surrounding a start codon and the distance from the cap structure. The context most suitable for AUG initiation has been shown to be GCCA/GCCAUGG (termed Kozak sequence),in which A or G at −3 and G at +4 (the A at the AUG codon being +1) are the most important nucleotides. Comparing with the Kozak sequence, all three uAUGs of *NRXN2α* are in an improper context for initiation. Since the uAUG1 of uORF1 is also located close to the cap and is thus less likely to initiate translation, we suspect that only a few ribosomes will initiate translation at uAUG1. Most of the ribosomes would pass it, by so-called leaky scanning, to initiate translation at the next AUG. This is also consistent with the results that the 91, 194, and 319 nt at 5 prime end of 5′UTR, including uAUG1, were successively deleted without significant change in activity. There are three uORFs in the 5′UTR of *NRXN2α*, and most ribosomes that may pass uAUG1 initiate translation on the next uAUG. After translation of the uORF, some ribosomes do not dissociate from the mRNA strand, but continue to scan for the next AUG to initiation translation, known as reinitiation. The efficiency of reinitiation is generally considered low^32^, which may be due to one of the mechanisms by which the uAUG and uORF inhibit initiation of main ORF translation. In addition, production of peptides by uORFs can reduce the initiation of translation of the downstream ORF by either stalling the ribosome at the end of the uORF or inhibiting the initiation at main ORF^32–34^.

In addition to uORF, it is reported that stable secondary structures play an important role in regulating the initiation of translation^25,35^. High GC content and a highly negative folding free energy (ΔG) level of a 5′UTR are often used as parameters for predicting 5′UTR RNA secondary structures. For the 5′UTR of *NRXN2α*, these regions are GC rich and have the potential to form stable stem loops which would inhibit translation. Furthermore, the mRNA secondary structures prediction algorithm Mfold^36^ suggests a very stable secondary structure with several stem loops. To elucidate which region of the 5′UTR of *NRXN2α* is responsible for the translational repression, we constructed several 5′UTR deletions plasmids. After transfection of plasmids containing deletions of the 5′UTR from its 5′ end, we observe that nucleotides 401–462 of the *NRXN2α-*5′UTR are important for translation inhibition. A possible explanation for this observation can result from the formation of G-quadruplex. Recently, there is growing evidence that canonical repeats of G-rich stretches in DNA or RNA can form stable G-quadruplex secondary structures, which are implicated in human telomere, oncogenic promoter and 5′UTR^37,38^. Since *NRXN2α-*5′UTR has a high GC content and contains multiple successive GGG, as predicted using QGRS (a software that is extensively applied for the prediction of G-quadruplex^24,25^), it is likely to fold into a stable G-quadruplex secondary structure (nucleotides 424–451). In the current study, we used Fluorescence Spectroscopy to validate the presence of G-quadruplex in *NRXN2α-*5′UTR^39,40^. To investigate the effect of the G-quadruplex structure on translation, we performed Dual-Luciferase reporter assays with luciferase mRNAs containing the wild type G-quadruplex (*NRXN2α*-5′UTR nucleotides 424–451) or mutated G-quadruplex motif. Dual-Luciferase reporter assays reveal a significant decrease in luciferase activity of the wild type G-quadruplex containing reporter. However, mutation of the G-quadruplex completely loses inhibitory effects. It can be inferred that G-quadruplex secondary structures are involved in translational regulation either by inhibiting the formation of the initiation complex or by inhibition of the scanning ribosome^41^.

The 5′UTRs in mRNAs encoding transcription factors, protooncogenes, growth factors and their receptors tend to be longer and have more uORFs,more uAUGs and more complicated secondary structures^20^. The characteristics of these mRNAs are the basis of translation regulation mediated by eIF2 phosphorylation. eIF2 phosphorylation suppresses general translation, but selectively stimulates the translation of these specific mRNAs, especially in response to certain external stimuli and cellular stresses. Control of protein translation is particularly important for synaptic plasticity underlying memory formation^42^. Many neuron-specific genes also have long 5′UTR with multiple short uORF and are regulated in same way by eIF2 phosphorylation. Therefore, some researchers proposed that neurons use some mechanisms of translational regulation similar to those activated by stress during memory formation^43^. Neurexins are important proteins involved in synaptic formation and control of synaptic plasticity underlying learning and memory processes, and is considered to play a central role in the entire trans-synaptic signaling network that controls synaptic properties^44^. As the structural basis of learning and memory, the formation and function maintenance of synapses are a changing process and are partly activity dependent.This process may depend on rapid changes in synthesis of synaptic proteins, including neurexins, especially in some local synapses^45^. The rapid changes in protein synthesis may involve rapid regulation of the translation of existing mRNA. The increase in UTR length of mRNA is thought to be an evolutionary consequence and the longest median length of mRNA 5ʹ UTRs is in humans^25^. Therefore, the 5′UTR of *NRXN2α* and its participation in the regulation of translation may play important roles for the function of synapse and learning and memory. Although we do not yet know the specific scenarios in which it works, this will be an important direction for future research. Although the vast variety of molecular species in synaptic cell adhesion molecules produced by extensive alternative splicing is thought to be the molecular basis of highly specific synapses, such diversity may not be sufficient to form specificity of trillions of synapses. The magnitude of synaptic molecular expression may also be involved in the formation of synapse specificity.

## Methods

### Plasmids constructions

Human *NRXN2α*(NM_138732.2) expression plasmid tagged with flag at the end of *NRXN2α* coding sequence (pcDNA3.1-NRXN2α-DYK) was purchased from Genscript and renamed as pN2aF. Firefly luciferase reporter plasmid p4P was generated by cutting SV40 promoter sequence from pGL3promoter(Promega) and inserted into pGL4.10(Promega) at XhoI/HindIII cutting sites. *Renilla* luciferase reporter plasmid p3PRluc was generated by amplifying coding sequence of *Renilla* luciferase from plasmid phRL-CMV(Promega) by PCR and replacing the coding sequence of firefly luciferase in plasmid pGL3promoter at HindIII/XbaI cutting sites. To construct *NRXN2α* expression plasmid with 5′UTR, the 5′UTR of 462 nt based NM_138732.2 was amplified by PCR from human genome and inserted into pN2aF before NRXN2α cDNA by homologous recombination according to the manual of ClonExpress MultiS One Step Cloning Kit(Vazyme Biotech) and named as pN2aF + 5U. To construct p4P + 5U, the 5′UTR was amplified using plasmid pN2aF + 5U as a template and the backbone of p4P + 5U was amplified using plasmid p4P as a template. The resulted DNA fragments were joined together by same manual as above homologous recombination to generate p4P + 5U, in which the 5′UTR of NRXN2α was inserted before coding sequence of firefly luciferase. The methods for constructing other plasmids and the corresponding primers are described in Supplementary Methods.

### Cell culture and transient transfection

SH-SY5Y (Human neuroblastoma), SK-N-SH (Human neuroblastoma), U-87MG (Human glioblastoma) and HEK293 (Human Embryonic Kidney) cell lines were cultured in Dulbecco’s modified Eagle’s medium (DMEM) supplemented with 10% fetal bovine serum(Gibco) and 1% penicillin-streptomycin at 37 °C in an incubator with 5% CO_2_. Before transient transfection, cells were seeded at 4.0 × 10^5^/ml (HEK 293 cell), 5.0 × 10^5^/ml (U-87MG cell),6.0 × 10^5^/ml (SH-SY5Y and SK-N-SH cell) in individual wells (6-well plate or 48-well plate) and cultured about 12–24 hours until 90% confluence. Transient transfection of different plasmids was carried out using lipofectamine 2000(Invitrogen) according to the supplier’s instructions.

### RNA extraction and reverse transcription

Total RNA was extracted from cells cultured in 6-well plate with improved one step method of guanidinium isothiocyanate and phenol(Bioteke). The kit (PrimeScript RT Reagent Kit with gDNA Eraser) of reverse transcription from Takara (including removal of probable residual DNA) was applied to synthesize the first-strand cDNA from 1 µg of the total RNA samples.

### Quantitative real-time PCR(qPCR)

qPCR was performed using 2 × QuantiNova SYBR Green PCR Master Mix(QIAGEN) according to the supplier’s protocol and conducted in CFX96 Thermal Cycler (Bio-Rad). Primers for detection of NRXN2α, firefly luciferase and GAPDH are NRXN2α-F(5′-GCTGAGTGTCCAAGCGATGAT-3′)/NRXN2α-R (5′-GTCCTCCGTGATAATGGGCAA-3′), Luc-F(5′-TTCGACCGGGACAAAACCAT-3′)/Luc-R(5′-GGGATGATCTGGTTGCCGAA-3′) and GAPDH-F(5′-CTCCTCCACCTTTGACGC-3′)/GAPDH-R(5′-CCACCACCCTGTTGCTGT-3′). For each sample, triplicates were analyzed with each primer set, and the amounts of mRNA expression were calculated using the comparative CT method. And the expression amount of transcription was normalized by GAPDH. For each sample, three independent transfected cell RNA extraction and reverse transcription reactions were performed, and each reaction was assayed in triplicate for qPCR. The mRNA levels of NRXN2α or firefly luciferase were normalized with GAPDH. Relative expression levels were calculated using CFX Manager3.1 software(Bio-Rad) and the ΔΔCt method and presented by 2^−ΔΔCt^.

### Western blot

Cells in 6-well plates were transfected with 2 μg of plasmid DNA per well. 24 hours after transfection cells were harvested and lysed in 100 µl of cell lysis buffer(Beyotime) supplemented with protease inhibitors (Roche).Cell lysates were separated on a 10–12% SDS Tris-glycine gel, transferred into PVDF membrane and blotted with primary and secondary antibodies. Expressed proteins were visualized by enhanced chemiluminescence and quantified using Quantity One. The relative amounts of proteins were normalized with control proteins. Following antibodies against DYKDDDDK (Cell Signaling Technology) to detect neurexin2α tagged with flag, β-actin(Sigma), firefly luciferase (Santa Cruz) and EGFP(Beyotime) were used as primary antibody, Horseradish peroxidase conjugated anti-mouse and anti-rabbit IgG antibodies(Perkin Elmer) were used as secondary antibody.

### Dual-luciferase assay

Cells were cultured in 48-well plate and co-transfected with 0.27 μg of different firefly luciferase constructs and 30 ng of *Renilla* luciferase vector p3PRluc per well. The p3PRluc serves as transfection control to normalize the transfection efficiency of firefly luciferase reporter constructs. Twenty-four hours after transient transfection, cells were harvested in the passive lysis buffer (Promega) and luciferase activities were measured using the dual-luciferase reporter assay system and Glomax luminometer according to the supplier’s protocol (Promega). The ratio of firefly/*Renilla* luciferase activities were calculated as the normalized reporter activity.

### PPIX-binding assay

The oligonucleotide pairs of DNA-T7G4F/DNA-T7G4R, DNA-T7G4M10F/DNA-T7G4M10R were synthesized and annealed to form double-stranded DNA templates of DNA-T7G4 and DNA-T7G4M10.The RNA fragments of G4 and G4M10 were prepared by *in vitro* transcription according to the manual of TranscriptAid T7 High Yield Transcription Kit (Thermo Scientific) and with DNA-T7G4 and DNA-T7G4M10 as templates, respectively. The resultant RNA fragments were dissolved in TE buffer (50 mM Tris-HCl, 1 mM EDTA, pH 8.5) and heated at 90 °C for 5 min and then cooled down to room temperature overnight to form the special structure. PPIX(Sigma) and KCl were also dissolved in TE buffer, respectively. Then, the solution of the RNA, PPIX(Sigma) and KCl were mixed together with the final concentration of the RNA and PPIX as 1 μM, K^+^as 100 mM. Finally, the mixtures were incubated in the dark for 2 h at room temperature to achieve structural changes and PPIX binding. The fluorescence intensity was measured by Synergy H1 micro-plate reader (BioTek). The excitation spectra were set at 410 nm and the emission spectra was from 550 to 700 nm^46,47^.

## Supplementary information

Supplementary Information

## Data Availability

The authors declare that all data supporting the findings of this study are available within the paper and its supplementary information files.

## References

[1] Sudhof TC (2008). Neuroligins and neurexins link synaptic function to cognitive disease. Nat..

[2] Sudhof TC (2018). Towards an Understanding of Synapse Formation. Neuron.

[3] Park D, Bae S, Yoon TH, Ko J (2018). Molecular Mechanisms of Synaptic Specificity: Spotlight on Hippocampal and Cerebellar Synapse Organizers. Molecules and Cell..

[4] Ushkaryov YA, Petrenko AG, Geppert M, Sudhof TC (1992). Neurexins: synaptic cell surface proteins related to the alpha-latrotoxin receptor and laminin. Sci..

[5] Tabuchi K, Sudhof TC (2002). Structure and evolution of neurexin genes: insight into the mechanism of alternative splicing. Genomics.

[6] Hata Y, Butz S, Südhof TC (1996). CASK: A Novel dlg/PSD95 Homolog with an N-Terminal Calmodulin-Dependent. J. Neurosci..

[7] Grootjans JJ, Reekmans G, Ceulemans H, David G (2000). Syntenin-syndecan binding requires syndecan-synteny and the co-operation of both PDZ domains of syntenin. J. Biol. Chem..

[8] Yamagata A (2018). Structural insights into modulation and selectivity of transsynaptic neurexin-LRRTM interaction. Nat. Commun..

[9] Craig AM, Kang Y (2007). Neurexin-neuroligin signaling in synapse development. Curr. Opin. Neurobiol..

[10] de Wit J (2009). LRRTM2 interacts with Neurexin1 and regulates excitatory synapse formation. Neuron.

[11] Chubykin AA (2007). Activity-dependent validation of excitatory versus inhibitory synapses by neuroligin-1 versus neuroligin-2. Neuron.

[12] Rowen L (2002). Analysis of the human neurexin genes: alternative splicing and the generation of protein diversity. Genomics.

[13] Treutlein B, Gokce O, Quake SR, Sudhof TC (2014). Cartography of neurexin alternative splicing mapped by single-molecule long-read mRNA sequencing. Proc. Natl Acad. Sci. US Am..

[14] Anderson GR (2015). beta-Neurexins Control Neural Circuits by Regulating Synaptic Endocannabinoid Signaling. Cell.

[15] Schreiner D, Simicevic J, Ahrne E, Schmidt A, Scheiffele P (2015). Quantitative isoform-profiling of highly diversified recognition molecules. eLife..

[16] Wilkie GS, Dickson KS, Gray NK (2003). Regulation of mRNA translation by 5′- and 3′-UTR-binding factors. Trends Biochemical Sci..

[17] Jackson RJ, Hellen CU, Pestova TV (2010). The mechanism of eukaryotic translation initiation and principles of its regulation. Nat. Rev. Mol. Cell. Biol..

[18] Pestova TV, Kolupaeva VG (2002). The roles of individual eukaryotic translation initiation factors in ribosomal scanning and initiation codon selection. Genes. Dev..

[19] Zhou W, Song W (2006). Leaky scanning and reinitiation regulate BACE1 gene expression. Mol. Cell. Biol..

[20] Araujo PR (2012). Before It Gets Started: Regulating Translation at the 5′ UTR. Comp. Funct. Genomics.

[21] Kozak M (2001). Constraints on reinitiation of translation in mammals. Nucleic Acids Res..

[22] Morris DR, Geballe AP (2000). Upstream Open Reading Frames as Regulators of mRNA Translation. Mol. and Cell. Biol..

[23] Wen Y (2009). Loss-of-function mutations of an inhibitory upstream ORF in the human hairless transcript cause Marie Unna hereditary hypotrichosis. Nat. Genet..

[24] Kikin O, D’Antonio L, Bagga PS (2006). QGRS Mapper: a web-based server for predicting G-quadruplexes in nucleotide sequences. Nucleic Acids Res..

[25] Leppek K, Das R, Barna M (2018). Functional 5′ UTR mRNA structures in eukaryotic translation regulation and how to find them. Nat. Reviews. Mol. Cell Biol..

[26] Hellen Christopher U.T. (2018). Translation Termination and Ribosome Recycling in Eukaryotes. Cold Spring Harbor Perspectives in Biology.

[27] Komar AA, Hatzoglou M (2011). Cellular IRES-mediated translation: the war of ITAFs in pathophysiological states. Cell Cycle.

[28] Kozak M (1999). Initiation of translation in prokaryotes and eukaryotes. Gene.

[29] Sonenberg N, Hinnebusch AG (2009). Regulation of translation initiation in eukaryotes: mechanisms and biological targets. Cell.

[30] Johnstone TG, Bazzini AA, Giraldez AJ (2016). Upstream ORFs are prevalent translational repressors in vertebrates. EMBO J..

[31] Somers J, Poyry T, Willis AE (2013). A perspective on mammalian upstream open reading frame function. Int. J. Biochem. Cell Biol..

[32] Hinnebusch AG, Ivanov IP, Sonenberg N (2016). Translational control by 5′-untranslated regions of eukaryotic mRNAs. Sci..

[33] Rahmani F (2009). Sucrose control of translation mediated by an upstream open reading frame-encoded peptide. Plant. Physiol..

[34] Ebina I (2015). Identification of novel Arabidopsis thaliana upstream open reading frames that control expression of the main coding sequences in a peptide sequence-dependent manner. Nucleic Acids Res..

[35] Pelletier J, Sonenberg N (1985). Insertion Mutagenesis to Increase Secondary Structure within the 5′ Noncoding Region of a Eukaryotic mRNA Reduces Translation Efficiency. Cell.

[36] Zuker M (2003). Mfold web server for nucleic acid folding and hybridization prediction. Nucleic Acids Res..

[37] Patel DJ, Phan AT, Kuryavyi V (2007). Human telomere, oncogenic promoter and 5′-UTR G-quadruplexes: diverse higher order DNA and RNA targets for cancer therapeutics. Nucleic Acids Res..

[38] Lipps HJ, Rhodes D (2009). G-quadruplex structures: *in vivo* evidence and function. Trends Cell Biol..

[39] Kong D-M, Ma Y-E, Guo J-H, Yang W, Shen H-X (2009). Fluorescent Sensor for Monitoring Structural Changes of G-Quadruplexes and Detection of Potassium Ion. Anal. Chem..

[40] Kozak M (1986). Point Mutations Define a Sequence Flanking the AUG Initiator Codon That Modulates Translation by Eukaryotic Ribosomes. Cell.

[41] Huppert JL, Bugaut A, Kumari S, Balasubramanian S (2008). G-quadruplexes: the beginning and end of UTRs. Nucleic Acids Res..

[42] Sossin WS, Costa-Mattioli M (2018). Translational Control in the Brain in Health and Disease. Cold Spring Harb. Perspect. Biol..

[43] Chesnokova Ekaterina, Bal Natalia, Kolosov Peter (2017). Kinases of eIF2a Switch Translation of mRNA Subset during Neuronal Plasticity. International Journal of Molecular Sciences.

[44] Sudhof TC (2017). Synaptic Neurexin Complexes: A Molecular Code for the Logic of Neural Circuits. Cell.

[45] Bellato HM, Hajj GN (2016). Translational control by eIF2alpha in neurons: Beyond the stress response. Cytoskeleton.

[46] Guo S, Lu H (2017). Conjunction of potential G-quadruplex and adjacent cis-elements in the 5′ UTR of hepatocyte nuclear factor 4-alpha strongly inhibit protein expression. Sci. Rep..

[47] Li T, Wang E, Dong S (2010). Parallel G-quadruplex-specific fluorescent probe for monitoring DNA structural changes and label-free detection of potassium ion. Anal. Chem..

