# Supplementary material for: Translational Inhibition of α-Neurexin 2
Source: Sci Rep. 2020 Feb 25;10:3403. doi: 10.1038/s41598-020-60289-8 (PMC7042298; doi:10.1038/s41598-020-60289-8)
Supplement: Supplementary file 1 — Supplementary Information [file 41598_2020_60289_MOESM1_ESM.pdf]

Translational Inhibition of α-Neurexin 2

Xiaoting Ding, Shasha Meng, Jiahong Zhou, Juan Yang, Hongmei Li and Weihui Zhou

Supplementary Methods

Plasmid construction

To construct 5U-uATG1m, two DNA fragments were amplified with corresponding primer pairs and DNA of p4P+5U as template. The resulted DNA fragments were joined together by homologous recombination according to the manual of ClonExpress MultiS One Step Cloning Kit (Vazyme Biotech).

To construct plasmid 5U-91/462, 5U-194-/462, 5U-319/462, 5U-401/462, 5U-1/110, 5U-1/194, 5U-1/319, 5U-uATG2m, 5U-uATG3m, p4P+5UG4M10, DNA fragments were amplified with corresponding primer pairs and DNA of p4P+5U as template, resulted PCR products were self-ligated to generate circle plasmids.

To construct plasmid 5U-uATG12m, 5U-uATG13m, DNA fragments were amplified with corresponding primer pairs and DNA of 5U-uATG1m as template, resulted PCR products were self-ligated to generate circle plasmids.

To construct plasmid 5U-uATG23m, DNA fragments were amplified with corresponding primer pairs and DNA of 5U-uATG3m as template, resulted PCR products were self-ligated to generate circle plasmids.

To construct plasmid 5U-uATG123m, DNA fragments were amplified with corresponding primer pairs and DNA of 5U-uATG13m as template, resulted PCR products were self-ligated to generate circle plasmids.

To construct plasmid 5U-111/193, DNA fragments were amplified with corresponding primer pairs and DNA of 5U-1/193 as template, resulted PCR products were self-ligated to generate circle plasmids.

To construct plasmid 5U-194/318, DNA fragments were amplified with corresponding primer pairs and DNA of 5U-1/318 as template, resulted PCR products were self-ligated to generate circle plasmids.

To construct plasmid 5U-319/400, DNA fragments were amplified with corresponding primer pairs and DNA of 5U-319/462 as template, resulted PCR products were self-ligated to generate circle plasmids.

To construct plasmid p4P+G4, p4P+G4M1, p4P+G4M2, p4P+G4M3, p4P+G4M4 and p4P+G4M5, DNA fragments were amplified with corresponding primer pairs and DNA of p4P as template, resulted PCR products were self-ligated to generate circle plasmids.

To construct plasmid EGFP+G4 and EGFP+G4M1, DNA fragments were amplified with corresponding primer pairs and DNA of pEGFPC2 as template, resulted PCR products were self-ligated to generate circle plasmids.

To construct plasmid 5U-401/462G4M2, 5U-401/462G4M3, 5U-401/462G4M4, 5U-401/462G4M5, DNA fragments were amplified with corresponding primer pairs and DNA of 5U-401/462 as template, resulted PCR products were self-ligated to generate circle plasmids.

To construct plasmid 5U-uATG123m+G4M2, 5U-uATG123m+G4M3, 5U-uATG123m+G4M4, 5U-uATG123m+G4M5, DNA fragments were amplified with corresponding primer pairs and DNA of 5U-uATG123m as template, resulted PCR products were self-ligated to generate circle plasmids.

All plasmids were confirmed by DNA sequencing. Corresponding primers were listed in following Table.

Plasmid name	Forward primer	Reverse primer

p4P+5U	P4P5UTR.F：TTTGCAAAAAGCTTGGCTGCAGGGATGCGGCGGCG	P4P5UTR.R：GCATCTTCCATGGTGGCGCCTACGGCGGCCCCGG
	P4PF:GCCACCATGGAAGATGCCAAAAA	P4PR: GCCAAGCTTTTTGCAAAAGCCTAGG
5U-uATG1m	uATG1F：TTTGCAAAAAGCTTGGCTGCAGGGGTGCGGCGGCG	P4P5UTR.R
	P4PF	P4PR
5U-uATG2m	uATG2F：TGCAAGGGAGCCCCGCTCAGCGC	uATG2R：GGCTCACAGTGCCACGGGCCCGG
5U-uATG3m	uATG3F：GCCCGCGGCTCTGAAGCCTGAGC	uATG3R：GTATGGGGCGGGAGGGGGCC
5U-uATG12m	uATG2F	uATG2R
5U-uATG13m	uATG3F	uATG3R
5U-uATG23m	uATG2F	uATG2R
5U-uATG123m	uATG2F	uATG2R
5U-91/462	+91/+462F:TTTGCAAAAAGCTTGGCACGCGGCGGGGAGGGAG	P4P5UTR.R
5U-194/462	+194/+462F:TTTGCAAAAAGCTTGGCAGCTTCTGGCGCCCCGAG	P4P5UTR.R:
5U-319/462	+319/+462F:TTTGCAAAAAGCTTGGCGCGGCCGGGCCCATGG	P4P5UTR.R:
5U-401/462	uATG3F:GCCCGCGGCTCTGAAGCCTGAGC	+401/+462R:GCCAAGCTTTTTGCAAAAGCCTAGGCCTCC
5U-1/110	717F:GCCACCATGGAAGATGCCAAAAACATTAAGAAGGGCCCAGCG	+1/+110R:TCTCTCCCTCCCCGCCGCGTCCCC
5U-1/193	717F	+1/+319R:GGGCGAGGCGCGCAGAGGCCCAGATG
5U-1/318	717F	+1/+319R
5U-111/193	+111/+193F:GGCGCGAGGCGGCAGACACCG	+401/+462R
5U-194/318	+194/+318F：CAGCTTCTGGCGCCCCGAGAACCAGGC	+1/+319R
5U-319/400	P4PF：GCCACCATGGAAGATGCCAAAAAC	+319/+400R:GCATGGGGCGGGAGGGGG
p4P+5UG4M10	5UTR-M10-F: AGACGAAGCCGAAGCCGCCGTAGGCGCCACCATGG	5UTR-M10-R: GGCTCTCGGCTCTCGCTCAGGCTTCAGAGCCGCG

p4P+G4	G4F:GGGCGGGGCCGGGGGCCACCATGGAAGATG	G4R:GGCCCCCGGCCCCCGCCAAGCTTTTTGCAAAAGCCTAG
p4P+G4M1	G4F	M1R:GGCCCCCGGCTCTCGCCAAGCTTTTTGCAAAAG
p4P+G4M2	G4F	M2R:GGCTCTCGGCCCCCGCCAAGCTTTTTGCAAAAGCCTAG
p4P+G4M3	M3F:GAGCGGGGCCGGGGGCCACCATGGAAGATG	G4R
p4P+G4M4	M4F:GGGCGAAGCCGGGGGCCACCATGGAAGATGCC	G4R
p4P+G4M5	M5F:GGGCGGGGCCGAAGGCCACCATGGAAGATGCC	G4R
EGFP+G4	EGFP+G4F: GGGCGGGGCCGGGGGCCACCATGGTGAGCAAG	EGFP+G4R: GGCCCCCGGCCCCCGACCGGTAGCGCTAGCGGATC
EGFP+G4M1	EGFP+G4F	EGFP+G4M1R: GGCCCCCGGCTCTCGACCGGTAGCGCTAGCGGATC
5U-401/462G4M2	401/462-M2F:GGGGGCCGAGAGCCGGGCGGGGCCGG	401/462-M1R:GCTCAGGCTTCAGAGCCGCGGGCGCC
5U-401/462G4M3	401/462-M3F:GGGCCGAGCGGGGCCGGGGC	401/462-M3R:CCGGCCCCCGCTCAGGCTTCAG
5U-401/462G4M4	401/462-M4F:CGGGCGAAGCCGGGGCCGCCGTAGGC	401/462-M4R:GCCCCCGGCCCCCGCTCAGGCTTC
5U-401/462G4M5	401/462-M5F:CGGGCGGGGCCGAAGCCGCCGTAGGCGCCACC	401/462-M4R
5U-uATGm123+G4M2	401/462-M2F	401/462-M2R:GCTCAGGCTTCAGAGCCGCGGGCGCC
5U-uATGm123+G4M3	401/462-M3F	401/462-M3R
5U-uATGm123+G4M4	401/462-M4F	401/462-M4R
5U-uATGm123+G4M5	401/462-M5F	401/462-M4R

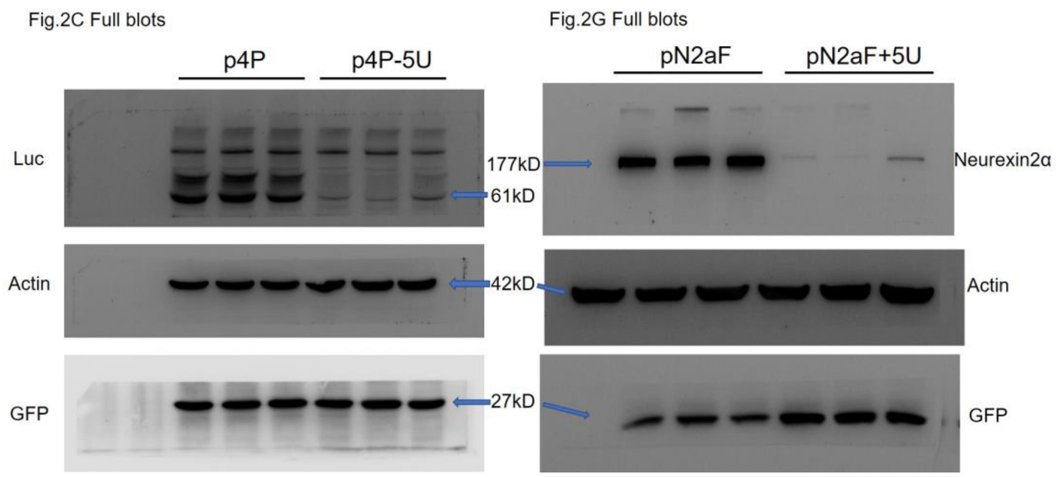

Supplementary Figure1. Full-length blots for figure 2.

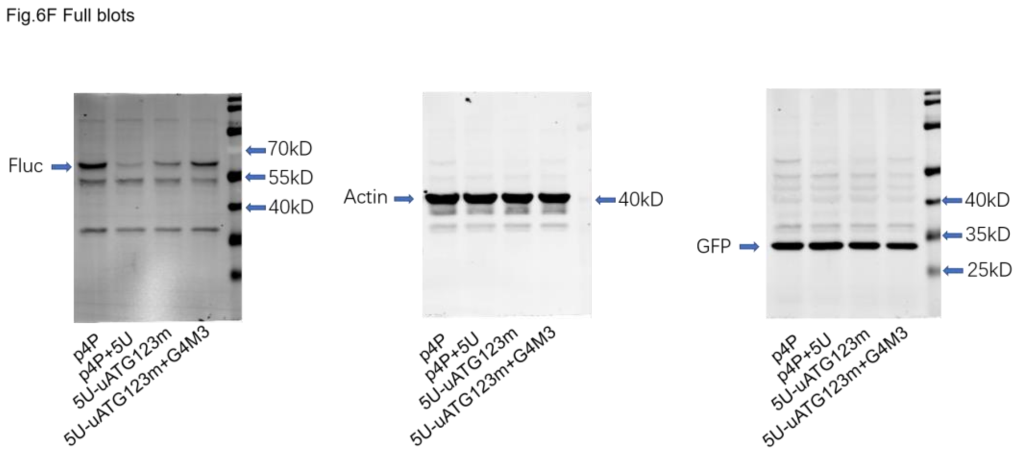

Supplementary Figure2. Full-length blots for figure 6F.
